# Supplementary material for: Transcriptomics Dissection of Calorie Restriction and Exercise Training in Brown Adipose Tissue and Skeletal Muscle
Source: Nutrients. 2023 Feb 20;15(4):1047. doi: 10.3390/nu15041047 (PMC9966723; doi:10.3390/nu15041047)
Supplement: Supplementary file 1 [file nutrients-15-01047-s001.zip › nutrients-2181632-supplementary.pdf]

Supplemental Figure and Figure legends

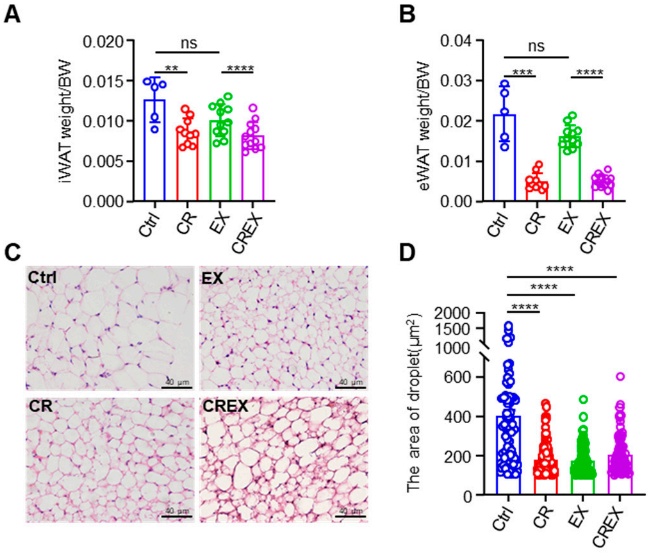

Figure S1. Phenotypic responses to CR with or without EX. (A) Ratio of iWAT weight to body weight in the four intervention groups. (B) Ratio of eWAT weight to body weight in the four intervention groups. (C) Representative H&E staining images of iWAT. scale bars, 40 μm (insets). (D)The area of droplet from H&E staining images of iWAT. Unpaired one-tailed t test or Wilcoxon rank sum and signed rank test was applied for statistical comparisons. Data are represented as mean ± SD. ** $p < 0.01$, *** $p < 0.001$, **** $p < 0.0001$; ns, not significant; BW, body weight; CR, calorie restriction; EX, exercise training; CREX, calorie restriction combined with exercise training; iWAT, inguinal white adipose tissue; eWAT, epididymal white adipose tissue.

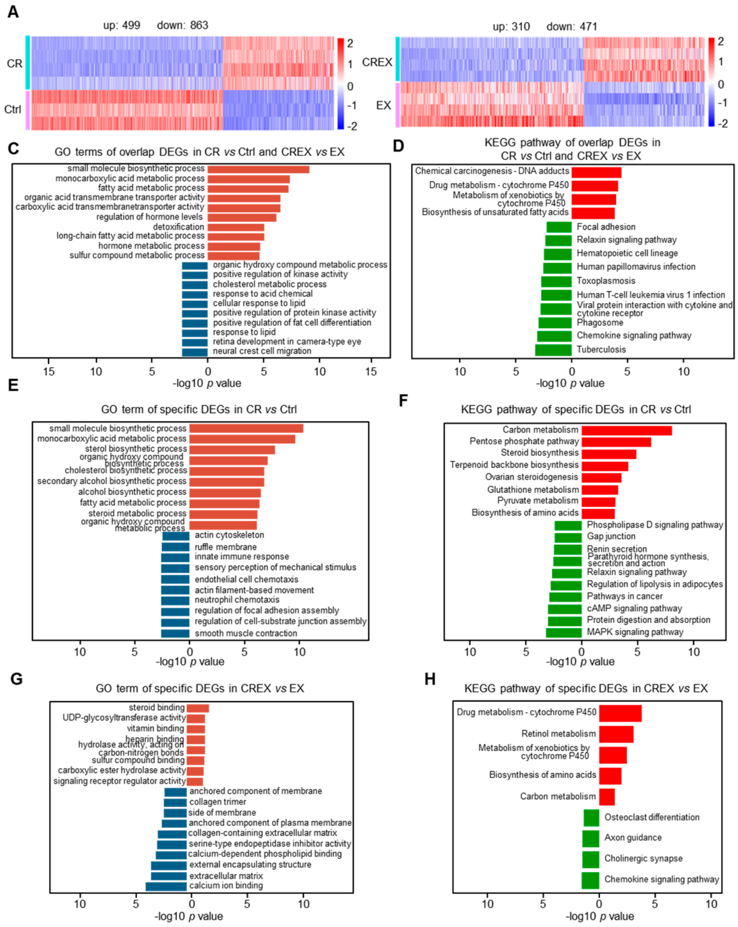

Figure S2. BAT exhibits transcriptomic changes upon CR with or without EX. Each dot indicates a sample. (A-B) Heatmap plot of DEGs in CR and Control group (A) and in CREX and EX group (B). (C) Enriched GO terms of common DEGs in the BAT upon CR with or without EX. (D) Enriched KEGG pathways of common DEGs in the BAT upon CR with or without EX. (E) Enriched GO terms of specific DEGs in the BAT upon CR without EX. (F) Enriched KEGG pathways of specific DEGs in the BAT upon CR without EX. (G) Enriched GO terms of specific DEGs in the BAT upon CR with EX. (H) Enriched KEGG pathways of specific DEGs in the BAT upon CR with EX. Brick red, enriched GO terms of upregulated DEGs; navy blue, enriched GO terms of downregulated DEGs; bright red, enriched KEGG pathways of upregulated DEGs; green, enriched KEGG pathways of downregulated DEGs.

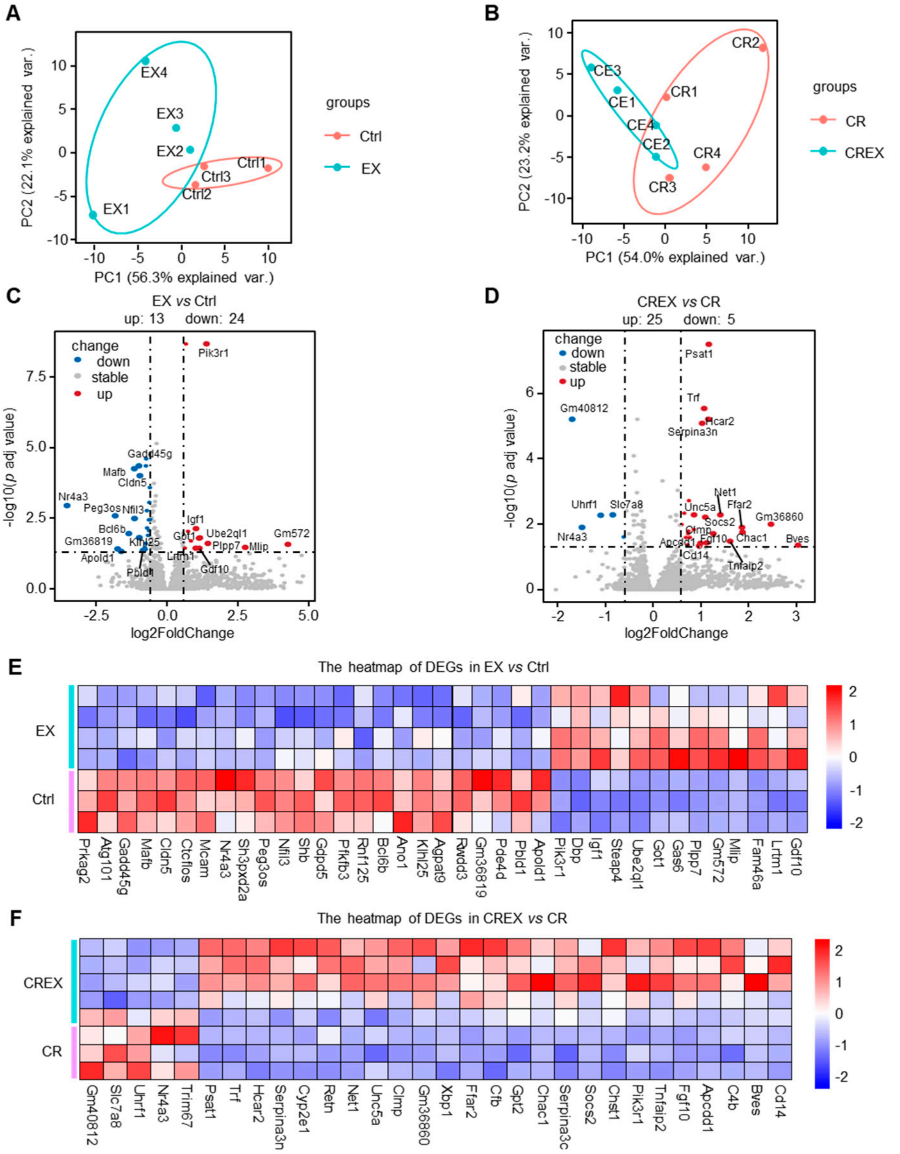

Figure S3. BAT exhibits transcriptomic changes upon EX with or without CR. (A-B) Principal component analysis plot of the BAT samples in Control vs EX group (A) and in CR vs CREX group (B). Each dot indicates a sample. (C-D) Volcano plot of DEGs in EX vs Control group (C) and in CREX vs CR group (D). Top 20 genes with the largest absolute value of log2FC were labeled. (Down: $p < 0.05$ and log2FC < -0.58; Up: $p < 0.05$ and log2FC > 0.58). (E-F) Heatmap plot of all DEGs upon EX without (E) or with CR (F).

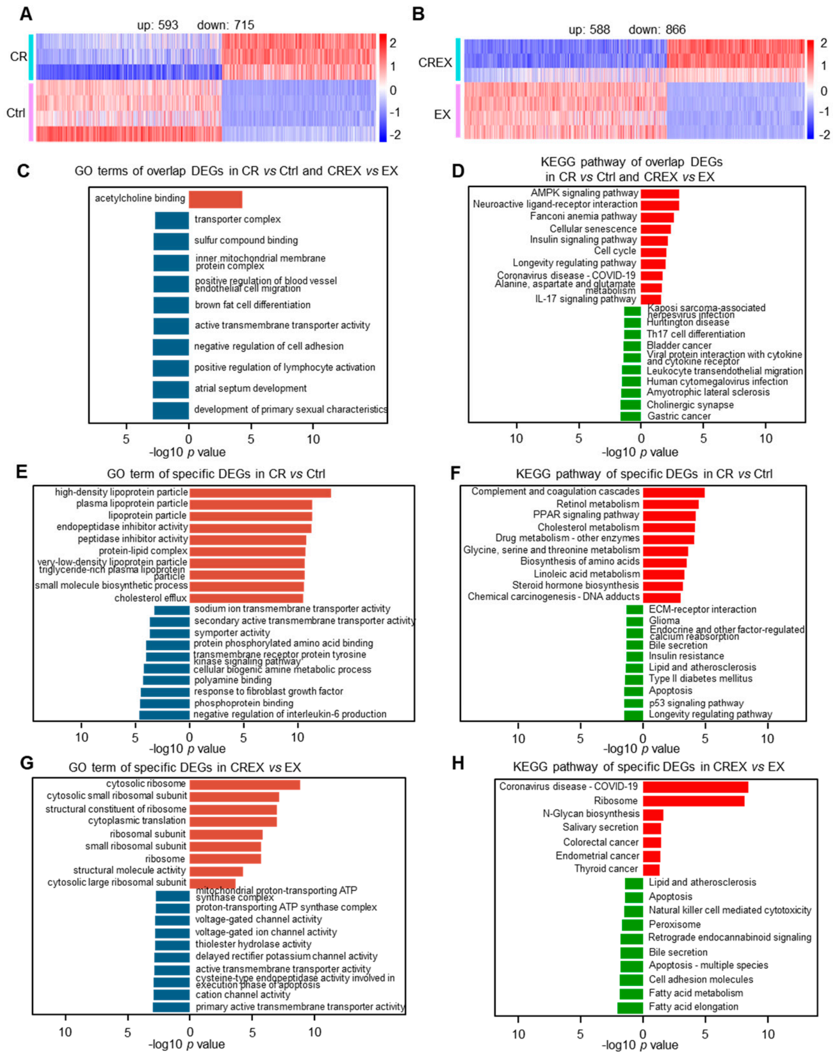

Figure S4. Skeletal muscle exhibits transcriptomic changes upon CR with or without EX. Each dot indicates a sample. (A-B) Heatmap plot of DEGs in CR vs Control group (A) and in CREX vs EX group (B). (C) Enriched GO terms of common DEGs in the skeletal muscle upon CR with or without EX. (D) Enriched KEGG pathways of common DEGs in the skeletal muscle upon CR with or without EX. (E) Enriched GO terms of specific DEGs in the skeletal muscle upon CR. (F) Enriched KEGG pathways of specific DEGs in the skeletal muscle upon CR. (G) Enriched GO terms of specific DEGs in the skeletal muscle upon CREX. (H) Enriched KEGG pathways of specific DEGs in the skeletal muscle upon CREX. Brick red, enriched GO terms of upregulated DEGs; navy blue, enriched GO terms of downregulated DEGs; bright red, enriched KEGG pathways of upregulated DEGs; green, enriched KEGG pathways of downregulated DEGs.

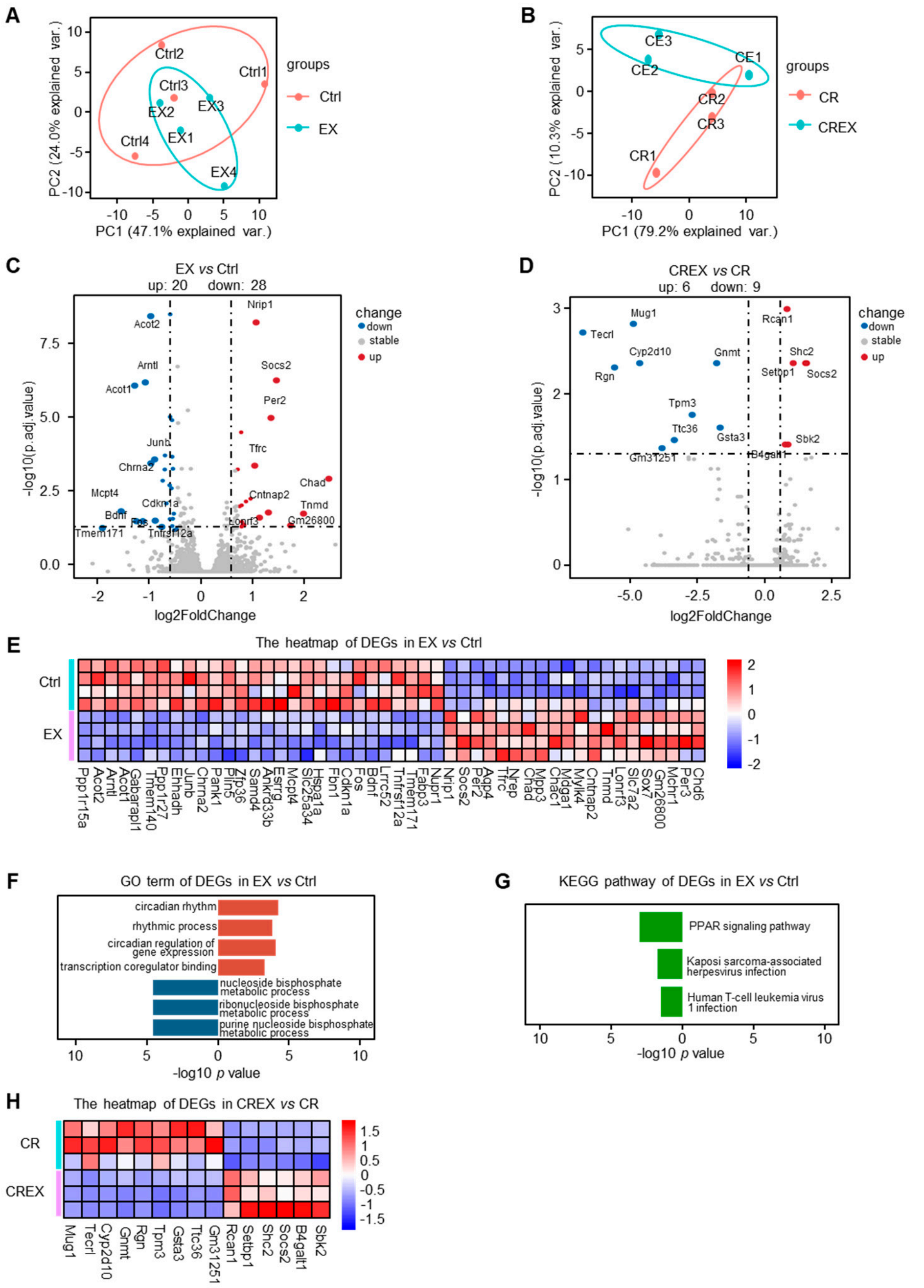

Figure S5. Skeletal muscle exhibits transcriptomic changes upon EX with or without CR. (A-B) Principal component analysis plot of the skeletal muscle samples in Control vs EX group (A) and in CR vs CREX group (B). Each dot indicates a sample. (C-D) Volcano plot of DEGs in EX vs Control group (C) and in CREX vs CR group (D). Top 20 genes with the largest absolute value of log2FC were labeled. (Down: $p < 0.05$ and log2FC < −0.58; Up: $p < 0.05$ and log2FC > 0.58). (E) Heatmap plot of all DEGs upon EX. (F) Enriched GO terms of all DEGs in the Skeletal muscle upon EX. (G) Enriched KEGG pathways of all DEGs in the Skeletal muscle upon EX. (H) Heatmap plot of all DEGs upon EX with CR.

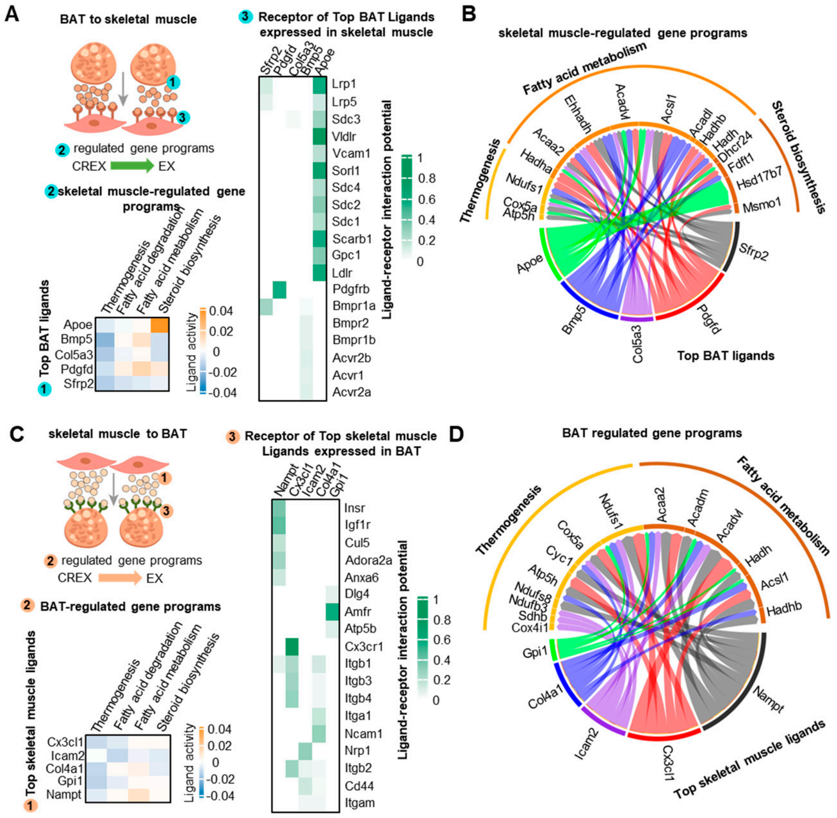

Figure S6. The intertissue communication between BAT and Muscle. (A) NicheNet workflow (top), the activity of top-five predicted BAT-derived ligands (bottom), and interaction potential for the predicted receptors in Skeletal muscle (right). (B) Computational interaction between BAT-derived ligands and their predicted Skeletal muscle target genes associated with the indicated KEGG pathways. (C) NicheNet workflow (top), the activity of top-five predicted skeletal muscle-derived ligands (bottom), and interaction potential for the predicted receptors in Skeletal muscle (right). (D) Predicted interaction between skeletal muscle-derived ligands and their predicated target genes in BAT related to the indicated KEGG pathway.
